# Pasteur and His Work
*" Pasteur and His Work." By L. Descour. Translated from the French by A. F. and B. H. Wedd, M.D. (Fisher Unwin. 15s. net.)


**Published:** 1922-08

**Authors:** 


### THE REVIEW OF THE MONTH.

### PASTEUR AND HIS WORK.*

OF the host of visitors to Paris, there are but few who make a visit to the somewhat sombre Rue d'Ulm. Yet it is a pilgrimage well worth the making to do honour to a benefactor to the world for all time and one of the most famous sons of France—Louis Pasteur. Affixed to a wall facing the street is a stone slab upon which may be read: "Here was the Laboratory of Pasteur." Then follows a list of his chief contributions to science with their dates: "1857, Fermentations; 1860, Spontaneous Generation; 1865, Diseases of Wine and Beer; 1868, Diseases of Silkworms; 1881, Virus and Vaccines; 1885, Prophylaxis Against Rabies." Yet these are but some of the milestones along the road of scientific investigation and discovery, which throughout a long life Pasteur pursued without faltering. In addition to the benefits which he thus bestowed upon humanity, there must be added the indirect but incalculable influence which he exerted by establishing a new school of scientific thought, and by training collaborators in his own exact methods of research, who have handed on the lamp of learning and have shed further lustre upon the Institut Pasteur—Roux, Yersin, Metchnikoff and the rest. Yet, though he lived to see the successful results of his labours and to receive the honours richly deserved, but only too often posthumously given, his path had sometimes been no easy one. Science, whether pure or applied, has ever been the Cinderella of all the works of public utility, and rated with but scant consideration by ministries and politicians. His experience for many of the most fruitful years of his life was no exception. When, for example, he applied to the Ministry of Public Instruction for a grant for his laboratory, he received the reply: "There is no heading under Expenditure which will permit me to allow you even 50 centimes towards the cost of experimental work." His own pocket supplied the necessary funds. At this time his assistant's stipend was the magnificent sum of 47.50 francs per month with board and lodging.

The chronological sequence of his researches is important, for each was a necessary preliminary to its successor. As a student of chemistry he had turned his attention to certain crystalline forms of tartaric acid and the tartrates, in which he made several important discoveries. Investigation of these questions led him almost inevitably to the consideration of fermentation and the production of alcohol through the agency of yeast, hitherto regarded as a purely chemical process. Pasteur proved that it was an organic change, and that "alcoholic fermentation is to be correlated with the life and the formation of the globules (of yeast) and not with their death or putrefaction." The question of spontaneous generation thus naturally arose in his mind. It is difficult for us to-day to realise the enormous importance to his subsequent discoveries that was exercised by his study of this question, or the obstinacy with which the doctrine of spontaneous generation was maintained until it was driven by the unequivocal force of experiment to the limbo of exploded dogmas. This question, once and for all time satisfactorily settled, opened up the whole field of the infections. He held the key which solved the problems of silkworm disease; anthrax, swine fever, rabies and the prevention of infections by appropriate vaccines. It was to Pasteur's early training in chemistry that his success in the solution of these questions was largely due, for it had taught him to attack the problems of living matter with the same rigidity of exact experiment which is necessary for one of the physical sciences. No precaution was too minute, no detail too meticulous to insure the validity of his deductions. Slow as he was to come to any conclusion without a thorough exploration of the grounds upon which he based his opinions, he was ever a doughty fighter against unsound arguments, inconclusive experiments or vague verbiage. Above all, his chief characteristic was an abiding enthusiasm for the honour and well-being of his country. During the Franco-German War he returned the Diploma of the Doctorate which had been awarded to him by the University of Bonn, with these words: "The sight of this parchment is now hateful to me, and I feel it an insult to have my name asociated with one doomed from henceforth to the execration of my countrymen, that of *Rex Gulielmus*."

Yet there was nothing of harshness in his outlook on life, and he was essentially sympathetic and human in his relation with those who came in contact with him. His humility and naïve simplicity are well seen in the characteristic letter which he wrote to Mme. Laurent to plead his suit for the daughter who afterwards became his wife: "I am afraid that Mlle. Marie attaches too much importance to first impressions, which can only be unfavourable to me. There is nothing in me to attract a young girl, but memory tells me that when people have known me well, they have liked me."

His whole life shows him to have been a singularly lovable personality, of which a strong devotion to his family and to his country, a true piety, humility, and a love of truth were the guiding principles. Apart from the application by Lister to the problem of surgical sepsis of the principles enunciated by him, which in its ultimate value to the human race transcends all his other discoveries, the material benefits which he conferred upon France and the whole world are incalculable. Ten years after the disastrous conflict with Germany, Huxley was able to state, without exaggeration, at a meeting of the Royal Society, "Pasteur's discoveries have brought France more than the five milliards of indemnity paid to Germany after the war of 1870."

*"Pasteur and His Work." By L. Descour. Translated from the French by A. F. and B. H. Wedd, M.D. (Fisher Unwin. 15s. net.)

M. Descour's book deals with the scientific side of Pasteur's work and must be read in conjunction with the fascinating "Life" by his son-in-law, M. René Vallery-Radot, to obtain a true perspective of his personality and character. In presenting the present book to the Academy of Medicine, Dr. Roux, one of Pasteur's most notable assistants, stated the truth when he said: "In a volume of less than 300 pages, the author has been able to relate what no one should be ignorant of in Pasteur's life, and what all who are engaged in scientific work should know of the achievements of this great man."—G.A.A.

